# CHD3 facilitates vRNP nuclear export by interacting with NES1 of influenza A virus NS2

**DOI:** 10.1007/s00018-014-1726-9

**Published:** 2014-09-12

**Authors:** Yong Hu, Xiaokun Liu, Anding Zhang, Hongbo Zhou, Ziduo Liu, Huanchun Chen, Meilin Jin

**Affiliations:** 1 State Key Laboratory of Agricultural Microbiology, Huazhong Agricultural University, Wuhan, 430070 People’s Republic of China; 2 College of Veterinary Medicine, Huazhong Agricultural University, Wuhan, 430070 People’s Republic of China; 3 Hubei Collaborative Innovation Center for Industrial Fermentation, Hubei University of Technology, Wuhan, 430070 People’s Republic of China; 4 College of Life Science and Technology, Huazhong Agricultural University, Wuhan, 430070 People’s Republic of China

**Keywords:** Influenza A virus, CHD3, Dense chromatin, VRNPs export

## Abstract

NS2 from influenza A virus mediates Crm1-dependent vRNP nuclear export through interaction with Crm1. However, even though the nuclear export signal 1 (NES1) of NS2 does not play a requisite role in NS2–Crm1 interaction, there is no doubt that NES1 is crucial for vRNP nuclear export. While the mechanism of the NES1 is still unclear, it is speculated that certain host partners might mediate the NES1 function through their interaction with NES1. In the present study, chromodomain-helicase-DNA-binding protein 3 (CHD3) was identified as a novel host nuclear protein for locating NS2 and Crm1 on dense chromatin for NS2 and Crm1-dependent vRNP nuclear export. CHD3 was confirmed to interact with NES1 in NS2, and a disruption to this interaction by mutation in NES1 significantly delayed viral vRNPs export and viral propagation. Further, the knockdown of CHD3 would affect the propagation of the wild-type virus but not the mutant with the weakened NS2–CHD3 interaction. Therefore, this study demonstrates that NES1 is required for maximal binding of NS2 to CHD3, and that the NS2–CHD3 interaction on the dense chromatin contributed to the NS2-mediated vRNP nuclear export.

**Electronic supplementary material:**

The online version of this article (doi:10.1007/s00018-014-1726-9) contains supplementary material, which is available to authorized users.

## Introduction

Influenza A virus (IAV) is the causative agent of seasonal influenza [1] and of the pandemics of 1918, 1957, 1968, and 2009 [2, 3]. It is a serious health threat with its capacity for cross-species transmission and potential to cause a 1918-like pandemic by strains such as avian H5N1 or H7N9 viruses [4, 5]. The eight segments of the RNA genome from IAV form ribonucleoprotein complexes (vRNP) with nucleoprotein (NP) and the trimeric polymerase. Unlike most RNA viruses, the transcription and replication of IAV take place in the nucleus [6]. Newly synthesized vRNPs exit from the nucleus into the cytoplasm [7] and assemble in the viral budding sites at the plasma membrane for the productive infection [8].

The nuclear export is a significant event in the viral life cycle. Both cellular and viral factors participate in the process. An important cellular factor for vRNP export is Crm1, a nuclear export receptor for proteins containing a characteristic leucine-rich nuclear export signal (NES) [9, 10]. In Crm1-dependent nuclear export, Crm1 forms a ternary complex with GTPase Ran and GTP in the nucleus for trafficking protein through the nuclear pore complex to the cytoplasm [11–13]. Influenza protein NS2 (a.k.a. nuclear export protein or NEP) is essential for Crm1-dependent vRNP export [14]. Proteolytic sensitivity analysis suggests that the C-terminus of the NS2 protein adopts a relatively rigid conformation, and that the N-terminus that contains two NESs (NES1 and NES2 [15]) has a more exposed conformation [16]. Before the new NES2 was discovered, NES1 was the only NES in NS2 and contradictory conclusions can be drawn from the available data with regard to the key role of NES1 in Crm1-dependent vRNP export [17, 18]. On the one hand, this is why vRNPs nuclear export adopts a Crm1-dependent manner, whereas NS2–Crm1 interaction is NES1-independent [19]. So far, the issue has been resolved by the existence of NES2; that is, NS2–Crm1 interaction would occur through the NES2 binding with Crm1, even though the NES1 was deleted [15]. On the other hand, this is why mutation at the crucial residues in the NES1 sequence in NS2 did not abolish the interaction between NS2 and Crm1 but significantly affected the vRNP nuclear export executed by NS2 [19]. This contradiction is still unresolved and it raises the question as to whether there are other factors assisting the process through its interaction with NES1.

In the present study, CHD3 was identified as a novel host protein involved in the NS2-associated nuclear export of vRNP. Our data suggest that the interaction would assist in tethering NS2 and Crm1 to vRNP facilitating the export. In addition, since only after CHD3 interacted with NES1, would the vRNPs be exported, it suggests that the interaction would expose the otherwise hidden NES1 for nuclear export.

## Materials and methods

### Cells and viruses

COS-1 cells were cultured in RPMI 1640 complete medium (HyClone, Thermo Scientific, Beijing, China) supplemented with 10 % heat-inactivated fetal bovine serum (FBS; HyClone, Thermo Scientific, Quarantine, Australia). MDCK cells were cultured in Dulbecco’s Modified Eagle complete medium (with 10 % FBS) (DMEM) (HyClone, China).

The WD virus (named as “rNS1-wt” in reference [20]) was constructed locally [20]. Mutant virus [WD–NS2(L19S)] was constructed as WD except that the pHW2000–NS [NS2(L19S)] plasmid was used. The WD–Flag–NS2 virus, expressing an NS2 fused with a 2× Flag tag, was constructed using pHW2000–Flag–NS2 plasmid which was described in “Plasmids”. MDCK cells were used to determine the viral titer as plaque forming units (p.f.u.) [21]. Cultivation and infection procedures were conducted as described earlier [20].

### Plasmids

The NS2 open reading frame (ORF) from IAV (A/chicken/Hubei/327/2004) was cloned into the pGBKT7 (pGBKT7–NS2) as a bait for yeast two-hybrid (Y2H) screening, the pBIND (pBIND-NS2) for mammalian two-hybrid (M2H) screening, and the pGEX-6p-1 (pGEX–NS2) for a glutathione S transferase (GST) pull-down assay. Various truncations [NS2(1–53 amino acid (aa)), NS2(54–122aa), NS2(6–122aa), NS2(9–122aa), NS2(16–122aa), NS2(20–122aa), NS2(26–122aa)] and mutants [NS2(M14A), NS2(M16A), NS2(L19A), NS2(L21A), NS2(M14A/M16A), NS2(M16A/L19A), NS2(M14A/L19A), NS2(M16A/L21A), NS2(L19A/L21A), NS2(M16A/L19A/L21A), NS2(M14A/M16A/L19A), NS2(L19S)] were constructed by site mutation based on pBIND-NS2. The C-terminus of human CHD3 (cCHD3) was cloned into the pCMVtag2B (Flag-cCHD3) for producing Flag-tagged cCHD3 and pACT (pACT-cCHD3) for M2H assay. A nuclear localization signal (NLS, PKKKRKV) [22] was attached immediate upstream of Flag-cCHD3 for expressing Flag-NLS-cCHD3. Myc-tagged wild-type NS2 (Myc-NS2) and mutant NS2 [Myc-NS2(L19S)] expression plasmids were generated by inserting each ORF into the pCDNA3.1-Myc vector. NS2 and NS2(L19S) ORF was cloned into pDsRed1-C1 vector to produce red fluorescent protein-NS2 (RFP–NS2) and RFP–NS2(L19S) fusion proteins. The pHW2000-NS [NS2(L19S)] was generated by site-mutation, in which a T to C mutation was introduced at position nt 539, which would not change NS1 protein. pHW2000-Flag-NS2 was constructed as follows. Based on the pHW2000-NS plasmid, two silent mutations in the endogenous splice acceptor site (5′ ttccaggacata 3′) were introduced (5′ttcccgggcata 3′) to prevent splicing at this site without changing NS1 protein. A new splice acceptor site corresponding to nucleotides 459-527 of the NS segment, a 2× Flag sequence, the entire NS2 ORF and 3′ noncoding region were added successively after NS1 ORF (Fig. S1 in supplemental materials). All other pHW2000 plasmids used in this study had been generated previously [20]. The primer sets and detailed strategies for constructing these plasmids are available upon request.

### Antibodies

The rabbit anti-NS2 polyclonal antibody and mouse anti-NP monoclonal antibody were prepared previously locally. Other antibodies were as follows: anti-histone H3.1 (P30266), anti-Flag (m20008) and anti-GAPDH (M20006) from Abmart (Shanghai, China); anti-c-Myc tag (CW0089), FITC-conjugated anti-rabbit IgG (CW0113), cy3-conjugated anti-mouse IgG (CW0159), HRP-conjugated anti-mouse IgG (CW0102) and anti-rabbit IgG (CW0103) from CWBIO (Beijing, China); anti-Crm1 (611832) from BD Transduction Laboratories (Franklin Lakes, NJ, USA); anti-HMGB1 (2600-1), anti-TFIIB (3728-1), anti-tubulin-alpha (2871-1) and anti-CHD3 (2969-1) from EPITOMICS (CA, USA); and normal mouse IgG (sc-2025) from Santa Cruz Biotechnology (CA, USA).

### Y2H

The Matchmaker Two-hybrid System3 (PT3247-1; Clontech, Mountain View, CA, USA) was used to screen a human fetal brain cDNA library (Cat. No. 638869; Clontech, USA). All procedures followed the protocol from the producer (PT3183-1; Clontech, USA).

### M2H

Two fusion constructs, 0.4 µg pBIND and 0.4 µg pACT, and 0.2 μg of the reporter pG5luc were cotransfected using Lipofectamine 2000 (lot# 1266554; Invitrogen, Life Technologies, NY, USA) into COS-1 cells. Firefly luciferase activity was normalized to Renilla luciferase activity as the relative activity according to the protocol (Part# TM049; Promega, Madison, WI, USA).

### Co-immunoprecipitation (IP) and GST pull-down assay

At 36 h after expression of Flag-cCHD3, cells were lysed using lysis buffer [50 mM Tris (pH 8.0), 100 mM NaCl, 20 mM NaF, 50 mM KH_2_PO_4_, 1 % Triton X-100, 10 % glycerol, and 0.1 mM dithiothreitol (DTT)] containing the 1 mM phenylmethyl sulfonylfluoride (PMSF; Amerisco, OH, USA). Cell debris and high molecular weight DNA were removed by centrifugation at 10,000*g* for 30 min. For Co-IP, the supernatant of lysate was diluted in the binding buffer [50 mM Tris-HCl (pH 8.0), 100 mM KCl, 0.1 mM EDTA, 0.2 % NP-40, 2.5 % glycerol, and 1 mM DTT], and incubated with 1 μg of an anti-Flag tag antibody and A/G PLUS-agarose (Santa Cruz Biotechnology) for 3 h at 4 °C. The beads were washed and then resuspended in sodium dodecyl sulfate (SDS) loading buffer. The bound proteins were resolved via SDS-PAGE (polyacrylamide gel electrophoresis) and transferred to a nitrocellulose membrane for western blot assay with ECL illumination (LH149493; Thermo, IL, USA). For GST pull-down, the GST–NS2 and GST proteins were expressed in *Escherichia coli* BL21 (DE3) and bound to glutathione-Sepharose 4B (GE Healthcare, Fairfield, CT, USA) for 2 h at 4 °C, respectively. The same procedure as Co-ip was performed except that cell lysate was incubated with the beads for 6 h at 4 °C.

### siRNAs

Duplex small interfering RNAs (siRNAs) targeting CHD3 (5′ gcgugacagugaggaggaa 3′) (si-CHD3) [23] and a validated negative control siRNA (5′ uucuccgaacgugucacgu 3′) (si-NC) sharing limited sequence identity with the known genes were purchased from GenePharma (Shanghai, China). An amount 50 nM of the siRNA was used for transfection.

### Immunofluorescence assay

The cells were fixed with 4 % paraformaldehyde, permeabilized using 0.2 % Triton X-100, and incubated with primary antibody for 30 min, then second antibody for 40 min. Nuclei were stained with DAPI (Invitrogen). Samples were examined by confocal microscopy with the LSM510 system (Carl Zeiss, Oberkochen, Germany). Colocalization was analyzed using the software Image J (NIH).

### Subcellular fractionation

Subcellular fractionation was performed as described previously [24] with modifications. Briefly, 5 × 10^7^ COS-1 cells were used for cell fractionation. The cytoplasmic fraction (cyt), nucleoplasmic fraction (nuc), low-salt chromatin fraction (ch150) and high-salt chromatin fraction (ch500) were resolved in 100 μl of sucrose buffer, 70 μl of nucleoplasm extraction, 70 µl of nuclease incubation buffer and 70 µl of chromatin extraction buffer, respectively. After collecting the ch500, the remaining pellet was resuspended in 70 µl of sample buffer without bromophenol blue. The supernatant was saved as the >ch500 fraction. The relative quantity of the proteins in each fraction was analyzed using the software Image J (NIH).

### Statistics

The mean values ± standard deviation (SD) were calculated, and *p* values were obtained according to Student’s *t* test for paired data. Statistical significance was defined as *p* < 0.05.

## Results

### CHD3 is a cellular partner to interact with IAV NS2 protein

**Fig. 1:**
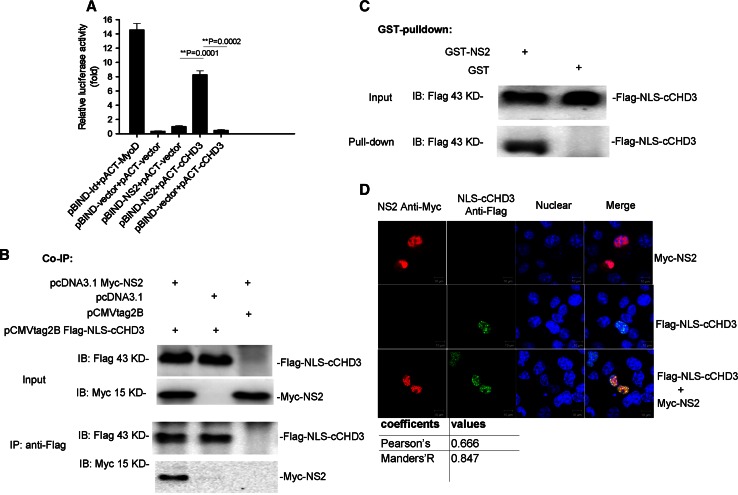
IAV NS2 protein interacts with CHD3. **a** NS2 binds CHD3 in M2H assay. COS-1 cells were cotransfected with the plasmids pACT–cCHD3, pBIND–NS2, and pG5luc, and then cell lysates were subjected to luciferase activity assays via M2H 24 h later. pBIND-Id and pACT-MyoD were used as positive controls, and pACT and pBIND as negative controls. The results are presented as the mean ± SD (**p < 0.01, *n* = 3). **b** NS2 interacts with CHD3 in Co-IP assay. Myc-NS2 and Flag-NLS-cCHD3 were cotransfected into COS-1 cells. 36 h after transfection, the cells were lysed, and immunoprecipitation was performed using an anti-Flag monoclonal antibody. The immunoprecipitated proteins were assayed with an anti-Myc polyclonal antibody. **c** NS2 interacts with CHD3 in GST pull-down assay. GST-tagged NS2 was expressed in bacteria. The cell lysates containing Flag-NLS-cCHD3 were mixed with beads to which GST-NS2 or GST alone was bound. The samples were analyzed using an anti-Flag monoclonal antibody. **d** NLS–cCHD3 interacted with NS2 in the nucleus by IFA assay. COS-1 cells on coverslips were transfected with plasmids coding the NLS–cCHD3 and NS2, and analyzed by IFA using antibodies against Myc (rabbit) and Flag (mouse). Colocalization was analyzed using Image J and the coefficients confirmed the strong colocalization

A human fetal brain cDNA library was screened by Y2H with NS2 as the bait to identify host proteins that interact with NS2. The C-terminus of human CHD3 (cCHD3) (aa 1667–2000, accession number: NM_001005273) was identified and its interaction with NS2 was confirmed in the M2H assay (Fig. 1a). Since cCHD3 has no native NLS, an NLS with the sequence of SV40 large T antigen [22] was fused to generate NLS–cCHD3 for mimicking the nuclear translocation of the endogenous CHD3. In Co-IP (Fig. 1b) and GST pull-down assays (Fig. 1c), it showed that, regardless of fusion of NLS with cCHD3, the NLS–cCHD3 could interact with NS2. Further, in transfection assay, although mostly located in the nucleus in COS-1 cells, NS2 exhibited a little cytoplasmic distribution (Fig. 1d, top). However, co-transfection with NLS–cCHD3 and NS2 resulted in a complete NS2 nuclear accumulation and a punctate distribution of NS2 similar to that of NLS–cCHD3 (Fig. 1d). The high co-localizing coefficients (Fig. 1d) assayed by Image J tool indicated that they were close enough in the spatial distance for the interaction to happen. Therefore, all the data above demonstrate that CHD3 is a cellular partner for the interaction with IAV NS2 protein and facilitates the NS2 nuclear accumulation.

### NS2–CHD3 interaction in vivo

**Fig. 2:**
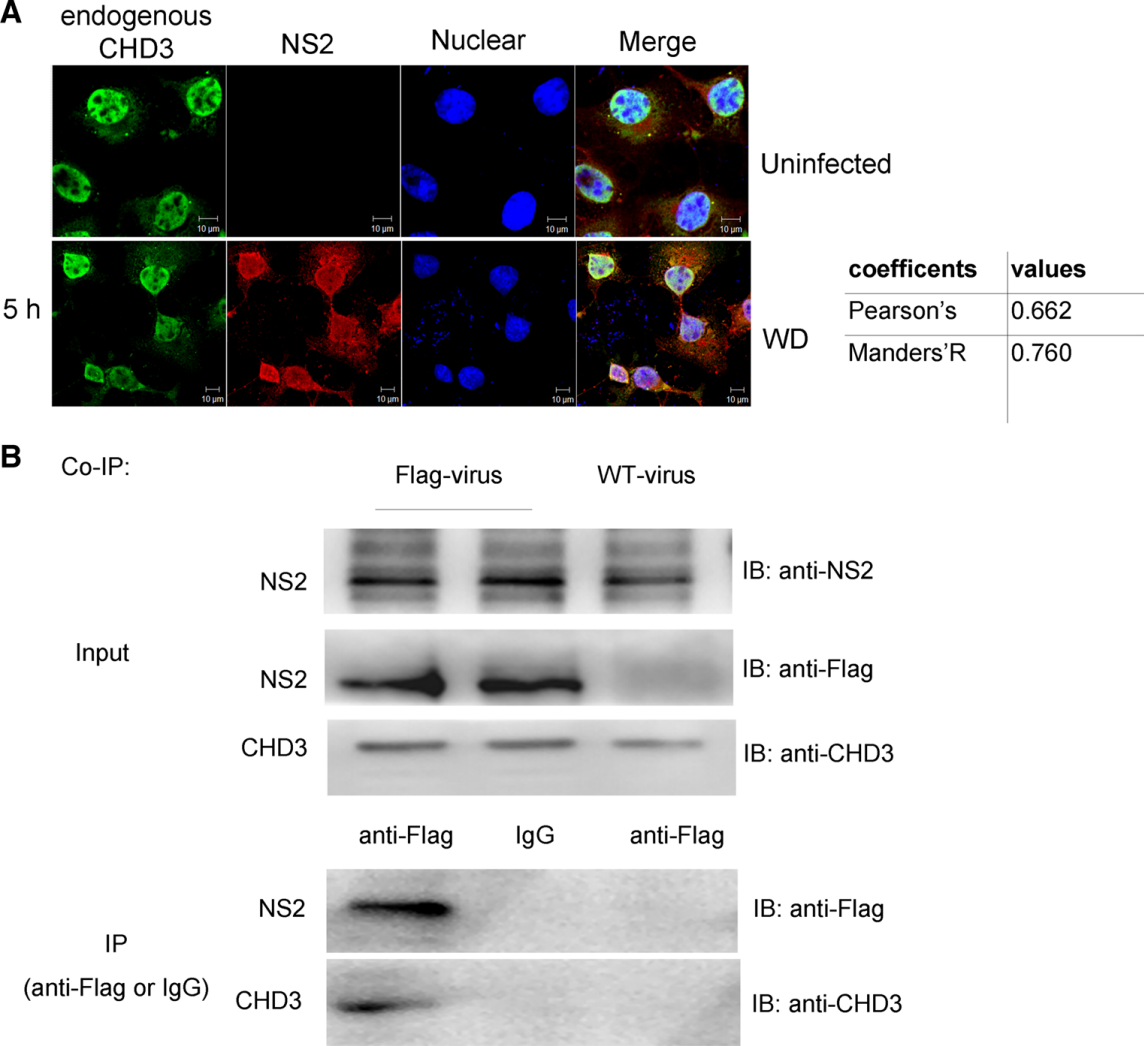
NS2 interacts with endogenous CHD3 under infection. **a** NS2 colocalizes with endogenous CHD3 under WD infection. COS-1 cells on coverslips were infected with WD [0.5 multiplicity of infection (MOI)], and at 5 h p.i., the cells were fixed for IFA with antibodies against NS2 and CHD3. **b** NS2 bound endogenous CHD3 during virus infection. COS-1 cells were infected by the WD or WD-Flag-NS2 viruses at an MOI of 3. At 8 h p.i., cell lysates were subjected to the IP assay with anti-Flag monoclonal antibody. The immunoprecipitated proteins were assayed with indicated antibody. Colocalization was analyzed using Image J and the coefficients confirmed the strong colocalization

To confirm the NS2–CHD3 interaction during virus infection, COS-1 cells were infected with WD virus. NS2 was shown to distribute throughout the nucleus. Though different from the punctate distribution of NLS–cCHD3, the endogenous CHD3 still predominantly co-translocated with NS2 in the nucleus in the early stage of the infection (Fig. 2a).

To further confirm the NS2–CHD3 interaction during virus infection in vivo, the recombinant IAV, WD-Flag-NS2 expressing the Flag-tagged NS2 protein, was generated (Fig. S1 in supplemental materials). CHD3 could be immunoprecipited with mouse anti-Flag monoclonal antibody with WD-Flag-NS2 infection (Fig. 2b), demonstrating that NS2 interacts with CHD3 in vivo.

### CHD3 interacts with the NES1 in NS2

**Fig. 3:**
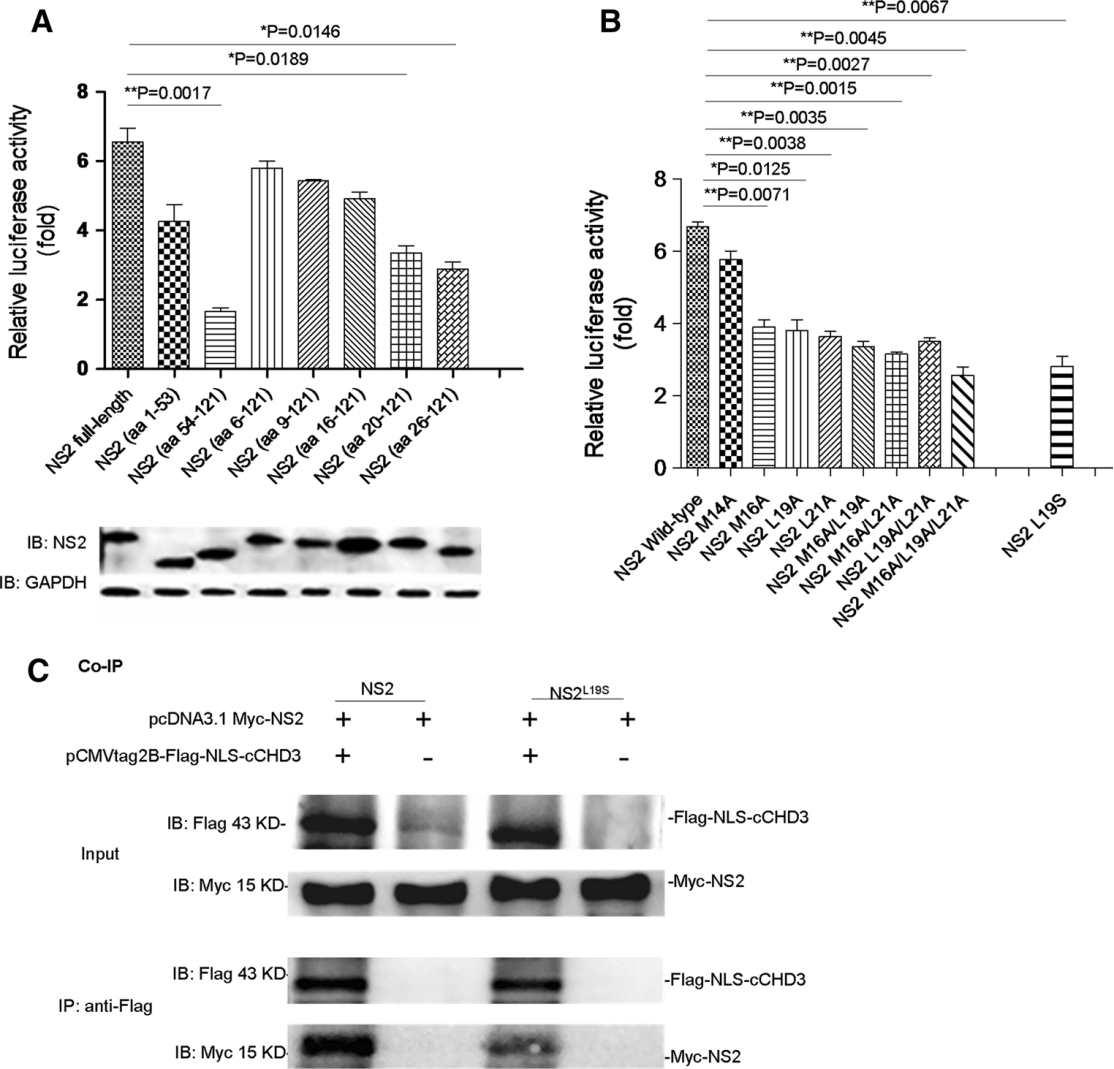
The key amino acids (M16, M19 and L21) of NS2 were involved in NS2–CHD3 interaction. **a** The NS2 N-terminus containing aa 16–20 mediated interactions with CHD3. *Top* COS-1 cells were cotransfected with pACT-cCHD3, pG5luc and pBIND-NS2 or pBIND-NS2-truncations. The strength of the interaction between cCHD3 and the NS2 truncations was assayed via M2H assay 24 h later. The interaction between CHD3 and the NS2 truncations was normalized to the self-activation of the NS2 truncations (co-transfection of the pBIND-NS2 truncations and pACT plasmid). The results are shown as the mean ± SD for three independent experiments (*p < 0.05, **p < 0.01, *n* = 3). *Bottom* the expression level of the NS2 truncations was detected with an anti-NS2 polyclonal antibody. GAPDH served as a protein loading control. **b** The NES of NS2 mediates interactions with CHD3. COS-1 cells were cotransfected with pACT-cCHD3, pG5luc and pBIND-NS2 or pBIND-NS2 mutants, and the strength of the interaction was assayed via M2H assay as above. The results are shown as the mean ± SD (*p < 0.05, **p < 0.01, *n* = 3). **c** NS2 (L19S) bound cCHD3 but weaker than NS2 did in Co-IP assay

To determine the interacting sequence in NS2, NS2 truncations, NS2(1–53aa) (with amino acid residues 54–121 deleted), NS2(54–121aa), NS2(6–121aa), NS2(9–121aa), NS2(16–121aa), NS2(20–121aa), and NS2(26–121aa), were prepared for analysis in M2H assay. The expression of the mutant NS2 proteins was similar to the wild-type levels (Fig. 3a, bottom). CHD3 interactions with truncation mutants of NS2(54–121aa), NS2(20–121aa), and NS2(26–121aa) were impeded (Fig. 3a, top), while the interactions with NS2(1–53aa), NS2(6–121aa), NS2(9–121aa), and NS2(16–121aa) were comparable to that with the full-length NS2(1–121aa) (Fig. 3a, top), indicating that the sequence in between amino acid residues 16–20 was important for the interaction.

Since the aa 16–20 is included in the NES1 motif (residues 12–21) in NS2 [15, 19], it is possible that the NES1 is responsible for the interaction. The key residues 16, 19, and 21 were then mutated [19] and it was shown that the single mutations (either M16A, L19A or L21A) or the triple mutation were detrimental to the interaction (Fig. 3b), indicating that NES1 is important in NS2–CHD3 interaction.

### Recruitment of Crm1 and NS2 to chromosome through the NS2–CHD3 interaction

**Fig. 4:**
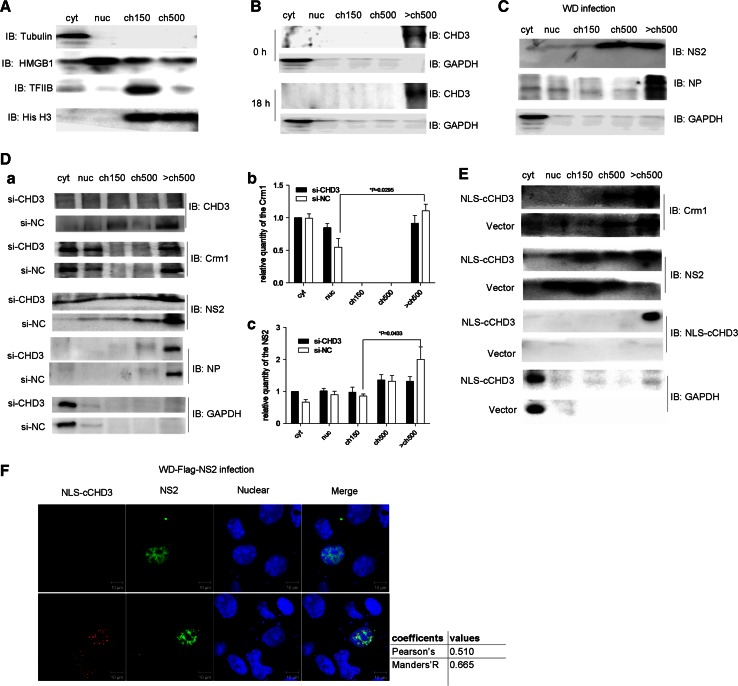
The NS2–CHD3 interaction played a significant impact on the distribution of Crm1 and NS2. **a** COS-1 cells were fractionated and cell fractionation was confirmed with antibodies against the following subcellular marker proteins: tubulin (cytoplasmic, cyt), HMGB1 (mainly nucleoplasmic, nuc), TFIIB (low-salt chromatin fraction, ch150) and histone H3 (whole chromatin fraction). **b** The distribution of endogenous CHD3 during WD infection. Cells were infected with WD virus (MOI 0.1). At 0 h and 18 h p.i., the cells were harvested and analyzed via western blotting. **c** The effect of WD infection (MOI 3) on the location of NP and NS2 at early stage of the infection (4 h p.i.). **d** *a* Knockdown of CHD3 using siRNA resulted in the diffusion of NS2 into all subcellular fractions and less Crm1 in the >ch500 fraction. COS-1 cells were transfected with si-CHD3 or si-NC; then, 24 h later the cells were infected with WD virus (MOI 3) for 4 h. The relative quantities of the NS2 (*b*) and Crm1 (*c*) in each fraction were analyzed using the software Image J (NIH) (*p < 0.05, **p < 0.01, *n* = 3). **e** NLS–cCHD3 changed the Crm1 and NS2 distribution under WD infection. COS-1cells were transfected with NLS–cCHD3 or empty vector as a control; 24 h later, the cells were infected with WD virus (MOI 0.3) for 10 h. **f** IFA assaying the effect of NLS–cCHD3 overexpression on location of NS2 and Crm1 at early stage of infection. COS-1 cells transfected with plasmid expressing NLS-Myc-cCHD3 (*top*) or untransfected (*bottom*) were infected with WD-Flag-NS2 virus. The location of NLS-Myc-cCHD3 and NS2 was marked by IFA. Colocalization was analyzed using Image J and the coefficients confirmed the strong colocalization. GAPDH was used to control the protein loading

It was shown that, during IAV infection, vRNPs export complexes along with NS2 and Crm1 were tethered to the host dense chromatin with very high affinity, which was essential for the NS2 and Crm1-dependent vRNP nuclear export [24]. It is possible that the NS2–CHD3 interaction is responsible for the recruitment of NS2 to the chromatin in this process. To confirm this, COS-1 cells were fractionated into cytoplasmic (cyt), nucleoplasmic (nuc), low-salt chromatin (ch150), high-salt chromatin (ch500), and very high-salt chromatin (>ch500) fractions (Fig. 4a) under infection for analyzing the distribution of CHD3, Crm1, NS2, and NP.

CHD3 was found in the >ch500 fraction, with or without the IAV infection (Fig. 4b). NP/vRNPs [9], and most NS2 were also in the >ch500 fraction at the early stage of IAV infection at 4 h p.i. (Fig. 4c). In contrast, when CHD3 was silenced by siRNA (Fig. 4d *a*), the amount of NS2 in >ch500 fraction was reduced at 4 h p.i. (Fig. 4d *a*), which was confirmed by a statistical test showing significant difference of NS2 content between ch150 and >ch500 in si-NC but not in si-CHD3 treatment (Fig. 4d *c*). In the experiment, it also showed that the distribution of Crm1 also changed with more Crm1 in nuc and less in >ch500 (Fig. 4d *a*), in which the significant difference of Crm1 between nuc and >ch500 was observed in si-NC but not in si-CHD3 sample (Fig. 4d *b*). The distribution of NP/vRNP was not altered with the knockdown of CHD3 (Fig. 4d).

It can be shown that recombinant NLS–cCHD3 was mostly associated with the chromatin under infection (Fig. 4e). The over-expression of NLS–cCHD3 also resulted in the association of the NS2 to the chromatin even at 10 h p.i. when NS2 would have distributed into other fractions. The punctuate distribution of NS2 in the nucleus was also observed with the transfection of NLS–cCHD3 and WD-Flag-NS2 infection (Fig. 4f). These data indicate that interaction with CHD3 would lead to the association of NS2, as well as Crm1 to the chromatin during IAV infection, which was suggested to be essential for the Crm1-dependent vRNP nuclear export [24].

### Modulation of vRNPs export by the NS2–CHD3 interaction

**Fig. 5:**
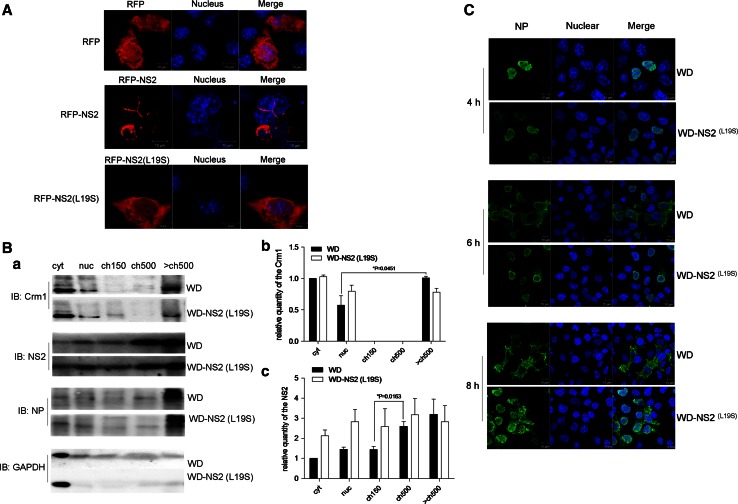
The NS2–CHD3 interaction modulated vRNP export. **a** L19S mutation interfered with the Crm1-dependent export activity of NS2. COS-1 cells on coverslips were transfected with plasmids coding the RFP, RFP–NS2 or RFP–NS2(L19S); 24 h later, the location of each expressing protein was collected using the confocal microscopy. **b** *a* The WD–NS2 (L19S) providing weakened NS2–CHD3 interaction exhibited less NS2 and Crm1 in >ch500. COS-1 cells were infected with WD–NS2 (L19S) or WD virus at MOI 3 for 4 h and cell fractionations was made immediately. The relative quantities of the NS2 (*b*) and Crm1 (*c*) in each fraction were analyzed using the software Image J (NIH) (*p < 0.05, **p < 0.01, *n* = 3). **c** The WD–NS2(L19S) delayed vRNPs export. COS-1 cells on coverslips were infected with WD–NS2(L19S) or WD virus at MOI 0.5. At 4, 6 and 8 h p.i., cells were fixed and permeabilized. vRNP localization was assessed via IFA using an anti-NP monoclonal antibody

It was shown that L19S mutation in NS2 would weaken the interaction with CHD3 (Fig. 3b, c). To indicate the role of NES1–CHD3 interaction in the nuclear export, the NS2 and NS2(L19S) were fused with RFP. This showed that, unlike the complete cytoplasmic location of RFP–NS2, the RFP–NS2(L19S) exhibited some weaker nuclear distribution (Fig. 5a), suggesting that positions 14–21 do have a degree of NES function. As the observation of delayed vRNP export by NES1 mutation during IAV infection [15, 19], it is curious that the mutation in NES1 did not reduce the interaction of NS2 with Crm1 but hindered the fusion export, although to a lesser extent. As an explanation, disruption of the NS2–CHD3 interaction by the NES1 mutation emerged as a way of interfering with NS2-mediated export function. A recombinant virus, WD–NS2(L19S), was generated with this mutation for the investigation. The overlapping NS1 was not changed in the resulting virus. It could be shown that, when compared with WD infection, at early stage of WD–NS2(L19S) infection, NS2 tended to enter into cyt, nuc and ch150 fractions, and less Crm1 and NS2 were in the >ch500 and ch500 fractions, respectively (Fig. 5b *a*). The observation were further supported by statistical analysis which showed significant difference of Crm1 between nuc and >ch500 (Fig. 5B, b), and significant difference of NS2 between ch150 and ch500 (Fig. 5b *c*) in WD but not WD–NS2(L19S) infection, confirming the role of NS2–CHD3 interaction in locating NS2 and Crm1 on dense chromation. Further, IFA showed that vRNP/NP was mostly distributed in the nuclei at 4 h p.i. in cells infected by either WD–NS2(L19S) or WD (Fig. 5c). At 6 h p.i., however, while the obvious export of vRNP could be observed under WD infection with NP translocation to the cytoplasm, WD–NS2(L19S) infection exhibited minimal vRNP export. At 8 h p.i., most vRNPs were exported under WD infection but few were under WD–NS2(L19S) infection. These data showed that the CHD3–NS2 interaction contributed to the export of vRNP.

### Modulation of IAV propagation by the NS2–CHD3 interaction

**Fig. 6:**
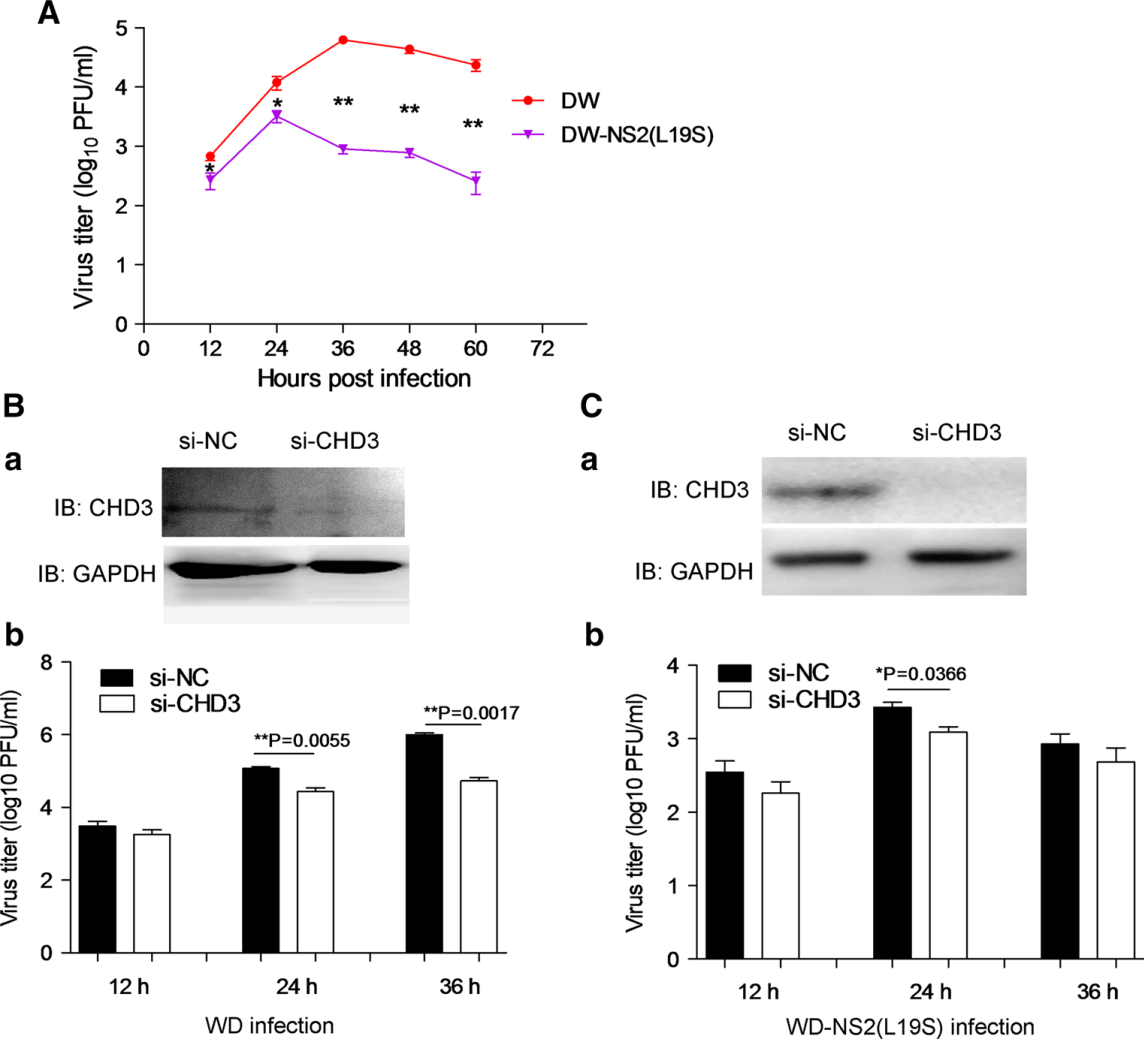
The NS2–CHD3 interaction modulated the propagation of the influenza A virus. **a** The WD–NS2(L19S) virus containing weaker NS2–CHD3 interaction exhibited poor propagation. COS-1 cells were infected and the supernatants were collected at the indicated time points for detecting the viral titer. **b** The knockdown of CHD3 (*a*) significantly decreased WD viral titers (*b*). **c** The knockdown of CHD3 (*a*) decreased WD–NS2(L19S) viral titers to a small extent (*b*). COS-1 cells were transfected with si-CHD3 or si-NC and infected with the WD or WD–NS2(L19S) virus (MOI 0.1) at the 24 h post-transfection. At 12, 24 and 36 h p.i., the cell supernatants were collected to measure the viral titer. The results are presented as the mean ± SD (*p < 0.05, **p < 0.01, *n* = 3)

Since vRNA export is an important step in the lifecycle of IAV, the interference on vRNA export may affect IAV propagation. It can be shown that WD–NS2(L19S), which encoded the mutant NS2 weakening the NS2–CHD3 interaction, propagated poorly at all time points (Fig. 6a). The peak titer of WD–NS2(L19S) was approximately 20 times lower than that of the WD (Fig. 6a). Furthermore, the continuous propagation of WD–NS2(L19S) was shorter than that of WD (Fig. 6a).

Similarly, the knockdown of CHD3 (Fig. 6b *a*) significantly decreased the propagation of the wild-type WD virus. The titers of virus were 4.3 and 18.8 times lower than the control at 24 and 36 h p.i., respectively (Fig. 6b *b*). In comparison, knockdown of CHD3 (Fig. 6b *a*) had less effect on the propagation of WD–NS2(L19S) virus (Fig. 6b *b*). Additionally, though NS2 has been shown to regulate polymerase activity [25], the NS2–CHD3 interaction did not affect polymerase activity and IAV replication (Fig. S2 in supplemental materials). Therefore, the NS2–CHD3 interaction facilitated IAV propagation through the promoting effect on vRNP export.

### CHD3 interacts with NS2 from other strains of IAV

**Fig. 7:**
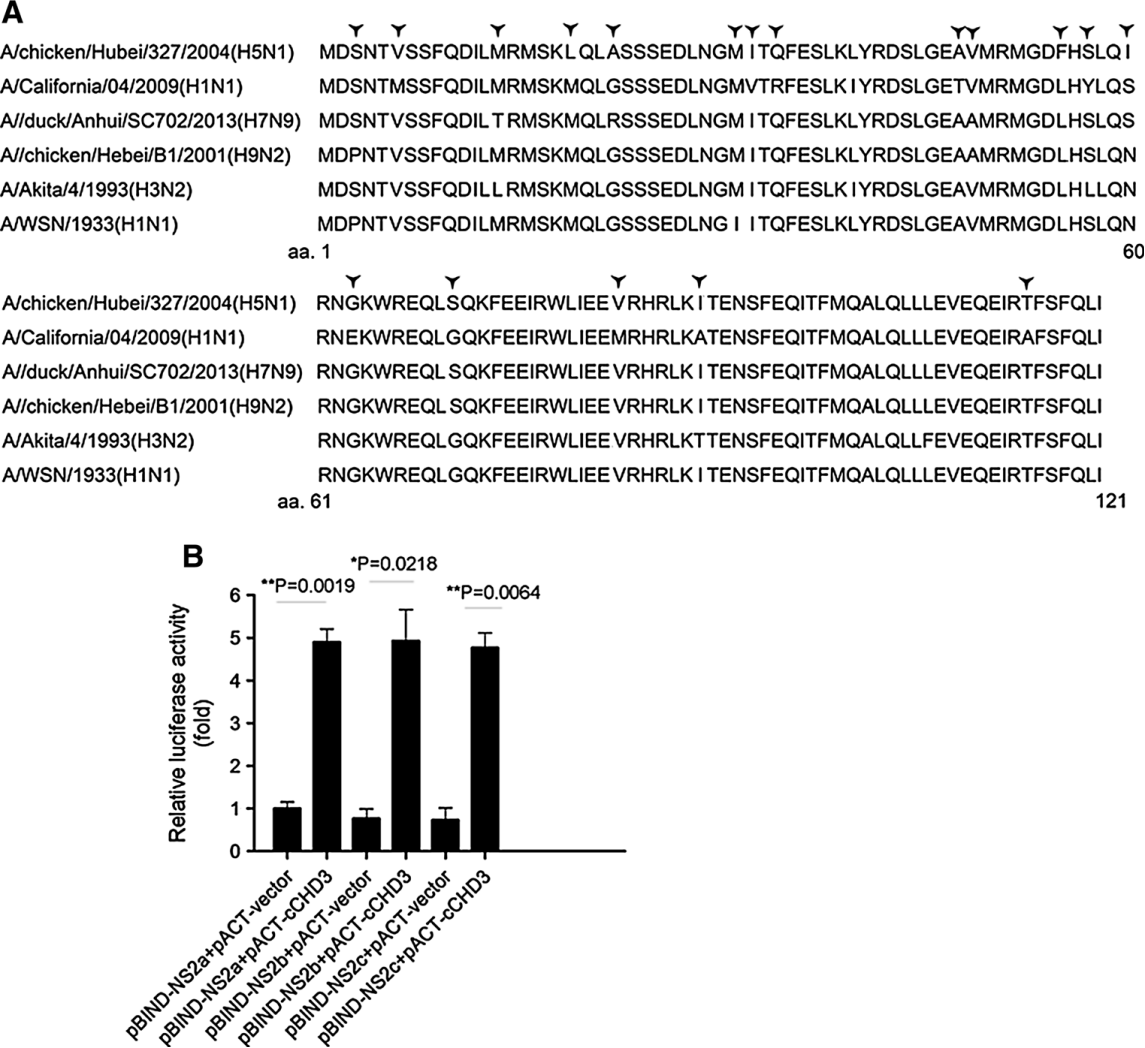
cCHD3 interacts with selected NS2 sub-types. **a** NS2s from different IAV were compared. *Star* indicates the different amino acid residue along the protein. **b** COS-1 cells were cotransfected with pACT-cCHD3, pG5luc and indicated pBIND-NS2 s sub-types. At 24 h post-transfection, luciferase activity assays were performed via M2H assay. The results are presented as the mean ± SD (*p < 0.05, **p < 0.01, *n* = 3). NS2a, A/WSN/1933 (H1N1); NS2b, A/duck/Hubei/W1/2004(H9N2); NS2c, A/Mexico City/001/2009(H1N1)

Selected strains of IAV were also tested for their NS2 interaction with CHD3. The NS2 of pandemic A/Mexico City/001/2009 (H1N1) virus, A/WSN/1933 (H1N1) virus, and avian influenza virus A/duck/Hubei/W1/2004 (H9N2) were employed in the M2H assay. The assay showed that all these NS2 could strongly interact with CHD3 (Fig. 7b), regardless of the amino acid differences alone the whole protein sequence (Fig. 7a).

## Discussion

IAV vRNPs display a biphasic pattern of intracellular localization during infection. They translocate in the nucleus to allow transcription to take place early in the life cycle of the virus, and are exported to the cytoplasm for package into progeny virions later in the life cycle. The current ‘‘daisy-chain’’ model indicates that during translocation of vRNPs, interaction between the IAV NS2 and Crm1 [26] is important for export of vRNPs to facilitate viral propagation. The translocation of vRNPs also depends on the function of NES1 in NS2. However, puzzling conclusions can be obtained from various experiments. On the one hand, NES1 would facilitate nuclear export dependent on Crm1 when it was fused onto other polypeptides [18], implicating a direct interaction with Crm1 as with most other NESs. On the other hand, previous study indicated that the NES1 in NS2 does not contribute to binding NS2 to Crm1; it was demonstrated that mutation on NES1 did not affect the interaction between NS2 and Crm1 [27], and leptomycin B treatment, an inhibitor to Crm1, has no effect on the localization of NS2 proteins [9]. Recently, it has been demonstrated that another leucine-rich NES2 is also involved in the nuclear export of vRNPs [15]. The interaction between NES2 and Crm1 may explain why deletion of NES1 does not abolish the NS2–Crm1 interaction. Nevertheless, despite all these studies, an unsettled question can still be raised as to why, in the case of NES1 mutation, NS2 by itself could not efficiently execute the export through its interaction with Crm1. At least we can rule out that the disruption of NES1 reduces the NS2–Crm1 interaction leading to the weaker nuclear export, since the disruption of NES1 actually enhanced the NS2–Crm1 interaction [27].

So, it seems that NES1 would not merely function through binding to Crm1. While the mechanism underlying the NES1 remains unclear, certain host partners can be proposed to interact with and mediate the NES1 function. As identified by our experiments, CHD3 could be an important host factor in vRNP export via interaction with the NES1 domain. From the data obtained, at least two possible mechanisms can be raised to interpret the role of the interaction in vRNP export.

1. The interaction would increase the export factors (NS2 and Crm1) around the vRNP, facilitating the export. It has been demonstrated that vRNPs were assembled on the chromatin before being exported to the cytoplasm [24]. Our data indicated that NS2 could be recruited to the dense chromatin via the NS2–CHD3 interaction. The subsequent NS2–Crm1 interaction would allow Crm1 preferential access to it, as was observed from the HIV-1 Rev protein [28]. The processes thus increased the export factors (NS2 and Crm1) around the vRNA on dense chromatin, which would aid vRNP export. Though it is technically difficult to observe the accurate subcellular localization for vRNPs, NS2, CHD3 and Crm1 at the same time, their colocalization on chromatin could be indirectly supported by the fact that the CHD3–NS2 interaction is important for tethering NS2 and Crm1 onto dense chromatin for vRNPs export. We should note that, since the construct with mutation in NS2 NES1 still apparently retains interaction with CHD3, the NES1 just seems required for maximal binding, which explains why the mutation in the NES1 only delayed but did not abrogate the export of vRNPs. Additionally, though NP would also be a candidate for recruiting Crm1 [9], our data indicated that NS2 should play a more important role in recruiting Crm1 than NP, because the shift of the location of Crm1 changed as did NS2 (Fig. 4d *a*).
2. The interaction would expose the NES1 for performing its function in vRNP export. During infection, it can be demonstrated that NS2 locates in the nucleus at an early stage (3.5 p.i.) (Fig. S3 in supplemental materials) and in the cytoplasm (5 p.i.) or cell membrane (6 p.i.) at later stages of the infection (Fig. S3 in supplemental materials). NS2 can also be shown to colocalize with NP/vRNP throughout the infection (Fig. S3 in supplemental materials). This implies that NES1 in NS2 could be hidden in the early stages of the viral life cycle and exposed to execute its function in the later stages. The data in the present study demonstrated that the vRNP export was executed only after the CHD3–NES1 interaction occurred. Thus, our data raised the possibility that the interaction between NES1 in NS2 and CHD3 on chromatin kept the NES1 exposed for nuclear export after vRNP assembly. Supporting this, when the NS2 was fused with the RFP, the NS2–RFP fusion protein instead completely located in the cytoplasm (Fig. 5a), in contrast to the whole cell distribution of RFP (Fig. 5a). However, when the L19S mutation was introduced into the NES1, which led to reduced interaction between NS2 and CHD3, NS2(L19S)–RFP relocated to some extent into the nucleus (Fig. 5a). The partial relocation of NS2(L19S)–RFP into nucleus would be explained by the fact that L19S mutation just reduced but did not abolish the NS2–CHD3 interaction (Fig. 3c). The role of the interaction in NES1 exposure could be illustrated using a monoclonal antibody against the NES1.

Previous research has shown that chromatin regions appear to be more dynamic than expected, indicating that heterochromatin is a platform which plays roles in the recruitment and spreading of regulatory proteins implicated in various aspects of chromosome biology [29]. Other studies have found the interaction of NP with nucleosomes [30] and the interaction of viral polymerase complex with CHD6 [31]. These would suggest the mechanism which could mediate the linkage of the subunits of vRNPs with dense chromatin. Further, the interaction of M1 with core histones 4 [32] was reported. Our findings additionally indicated the translocation of NS2 on dense chromatin at an early stage of infection. Therefore, all necessary subunits could be recruited to the dense chromatin, which supported that the chromatin was an excellent platform for IAV vRNPs assembly and its export.

In summary, this study identifies a new factor in NS2 mediated nuclear export. CHD3 can not only provide the conduit for the assembly of NS2 with vRNP on the chromatin but its interaction with NES1 in NS2 may also result in its exposure for Crm1-dependent nuclear export. Together with the existence of NES2, these observations consolidate the data from previously seemingly contradictory data and provide a crucial link in understanding the vRNP export during the life cycle of IAV.

## Electronic supplementary material

Below is the link to the electronic supplementary material.

Supplementary material 1 (TIFF 447 kb)

Supplementary material 2 (TIFF 497 kb)

Supplementary material 3 (TIFF 918 kb)

Supplementary material 4 (DOC 27 kb)

## Abbreviations

CHD3: Chromodomain-helicase-DNA-binding protein 3
cCHD3: C-terminus of human CHD3
GST: Glutathione S transferase
IAV: Influenza A virus
IFA: Immunofluorescence assay
IP: Immunoprecipitation
M2H: Mammalian two-hybrid
NEP: Nuclear export protein
NES: Nuclear export signal
Pfu: Plaque-forming units
siRNAs: Small interfering RNAs
Y2H: Yeast two-hybrid
WT: Wild-type
