# Supplementary figures and images for: CHD3 facilitates vRNP nuclear export by interacting with NES1 of influenza A virus NS2
Source: Cell Mol Life Sci. 2014 Sep 12;72(5):971–82. doi: 10.1007/s00018-014-1726-9 (PMC4323543; doi:10.1007/s00018-014-1726-9)

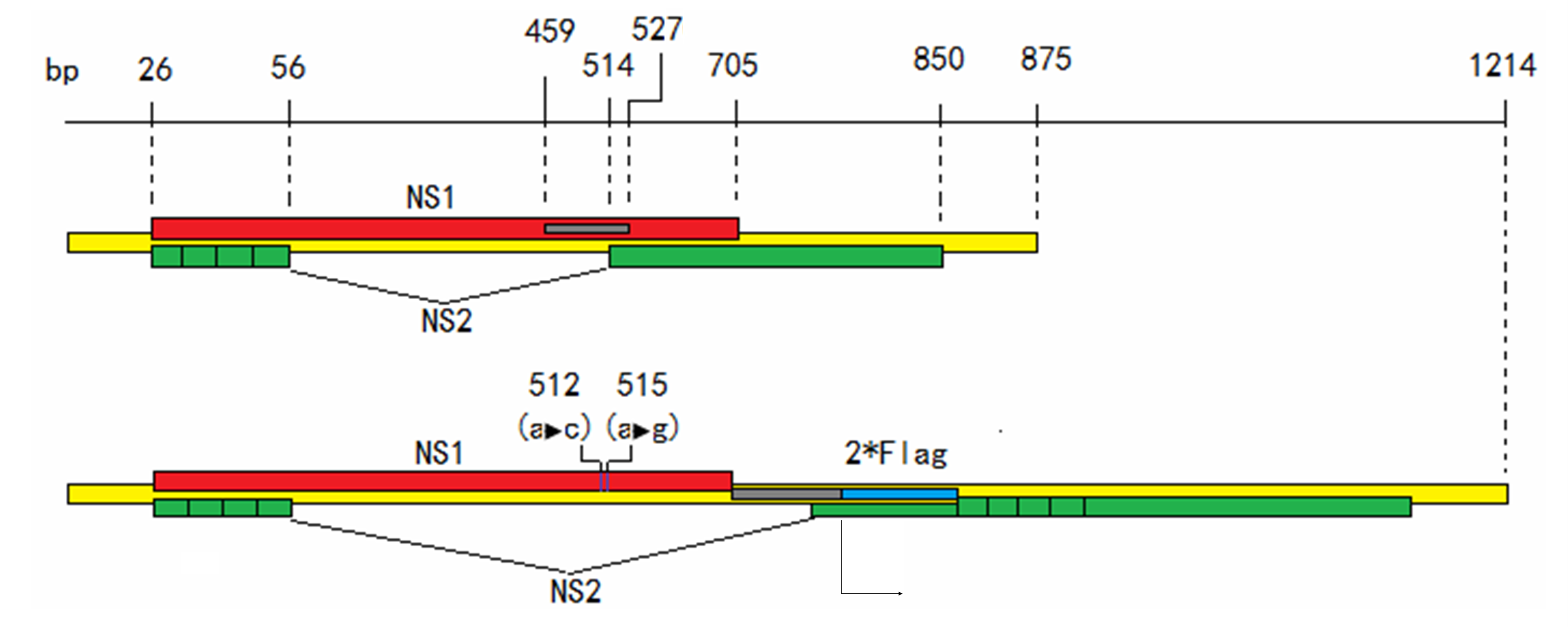

Supplement: Supplementary file 1 — Supplementary material 1 (TIFF 447 kb) [file 18_2014_1726_MOESM1_ESM.tif]

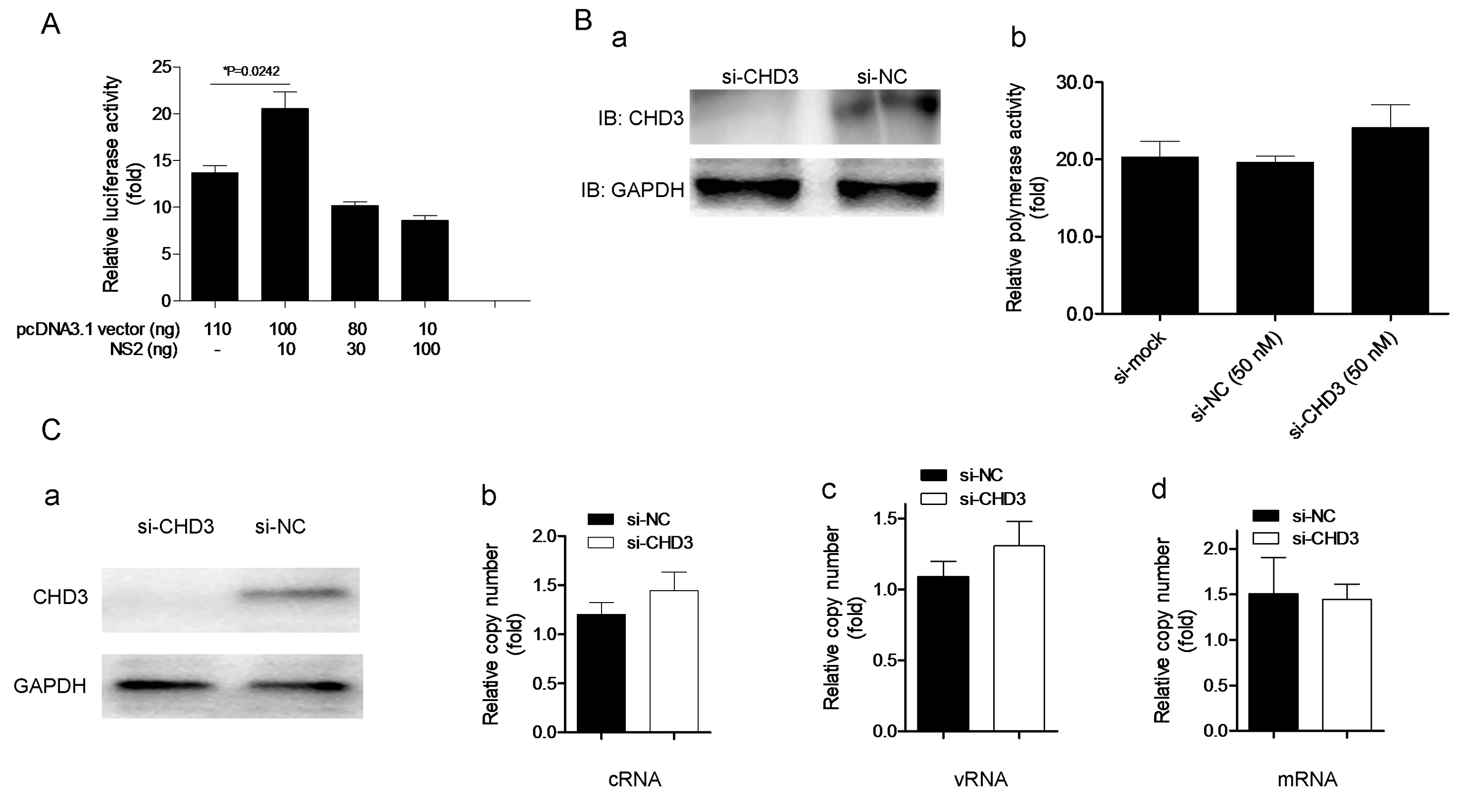

Supplement: Supplementary file 2 — Supplementary material 2 (TIFF 497 kb) [file 18_2014_1726_MOESM2_ESM.tif]

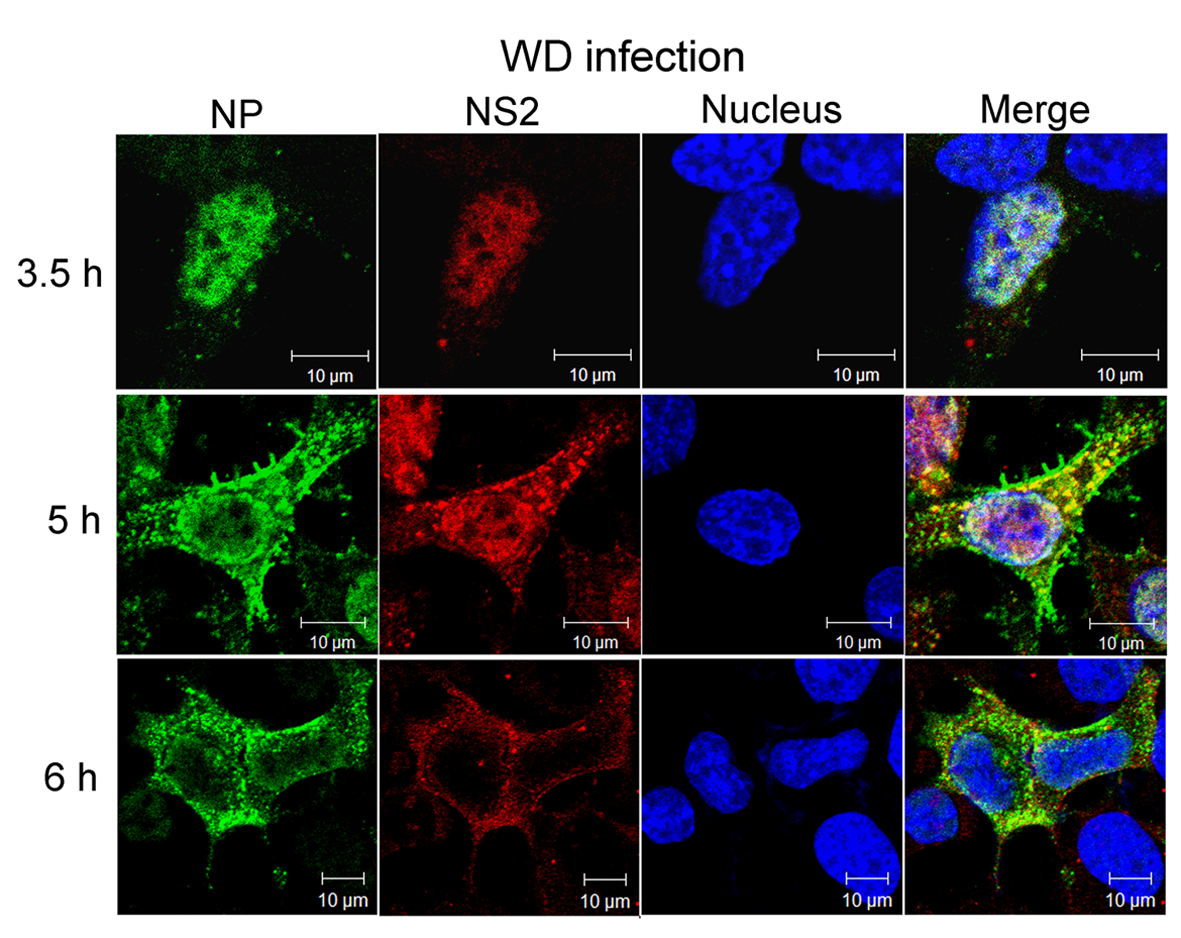

Supplement: Supplementary file 3 — Supplementary material 3 (TIFF 918 kb) [file 18_2014_1726_MOESM3_ESM.tif]
