# Supplementary material for: CHD3 facilitates vRNP nuclear export by interacting with NES1 of influenza A virus NS2
Source: Cell Mol Life Sci. 2014 Sep 12;72(5):971–82. doi: 10.1007/s00018-014-1726-9 (PMC4323543; doi:10.1007/s00018-014-1726-9)
Supplement: Supplementary file 4 — Supplementary material 4 (DOC 27 kb) [file 18_2014_1726_MOESM4_ESM.doc]

**Supplementary Information**

**Fig S1**

Construction of NS gene with Flag tag (Flag-NS2). On the sense strand, two silent mutations in the endogenous splice acceptor site in the NS1 ORF (5’ttccaggacata 3’) were introduced to prevent splicing at this original site (5’ttcccgggcata 3’), which would not change the amino acid residues of NS1. A new splice acceptor site corresponding to nucleotides 459-527 of the NS segment, a 2× Flag sequence, the entire NS2 ORF and 3’ noncoding region were added successively after NS1 ORF. Yellow represents NS gene; Red represents NS1; Green represents NS2; Gray represents the splice acceptor site corresponding to nucleotides 459-527 of the WT NS segment; Blue represents a 2x Flag sequence (DYKDDDDKDYKDDDDK); The nt 514 is the first base of the second exon of NS2.

**Fig S2**

The NS2-CHD3 interaction did not impair IAV replication. A, NS2 increased the polymerase activity in COS-1 cells at low concentration. B, Knocking down the CHD3 using siRNA (a) did not disturb the effect of NS2 on the polymerase activity (b) in COS-1 cells. The NS2 (10 ng) and siRNA (50 nM) were co-tranfected in to the cells. C, Knocking down the CHD3 using 50 nM siRNA (a) did not change the NP cRNA (b), vRNA (c), and mRNA (d) levels at early stage (4 h p.i.) during the WD infection (MOI 3) in COS-1 cells. The 'relative fold-change' refers to the ratio of amount of each RNA in siCHD3 and siNC sample. Total RNA in cells was extracted by using TRIzol (Invitrogen). Avian myeloblastosis virus (AMV) reverse transcriptase (Promega) and special primers for viral vRNA (5’ aaagcagggtagataat 3’), cRNA (5’ agtagaaacaagggtat 3’), and mRNA (5’ aacacaggaaacgctgagattgaa 3’) were used to generate cDNAs. The cDNA product (0.5 μl) was subsequently amplified in a 25 μl reaction mixture containing SYBR® Green Real-time PCR Master Mix (Roche, Indianapolis, IN, USA) and 0.2 μM each forward and reverse gene-specific primers (NP: forward 5’ gggccataaggaccagaagtg 3’, reverse 5’ cgcccctggaaagacacatc 3’). The data were assayed by relative Real-time PCR using ABI 7500.

**Fig S3**

A, The localization of NS2 and NP/vRNP during infection. COS-1 cells were infected with WD virus. At the indicated times, the cells were fixed and analyzed via IFA using an anti-NS2 polyclonal antibody and an anti-NP monoclonal antibody. The images were detected directly under confocal microscopy with the LSM510 system (Carl Zeiss, Germany). Colocalization was analyzed using the software Image J (NIH)
